# Quick and dirty or rapid and informative? Exploring a participatory method to facilitate implementation research and organizational change

**DOI:** 10.1108/JHOM-12-2020-0503

**Published:** 2021-09-22

**Authors:** Ulrica von Thiele Schwarz, Kin Andersson, Carina Loeb

**Affiliations:** Academy of Health, Care and Social Welfare, Mälardalen University , Västerås, Sweden; Department of Learning, Informatics, Management and Ethics, Medical Management Centre, Karolinska Institutet, Stockholm, Sweden; Academy of Health, Care and Social Welfare, Mälardalen University , Eskilstuna, Sweden

**Keywords:** Knowledge generation, Knowledge use, Implementation, Mode-1, Mode-2, Organizational change, Participatory

## Abstract

**Purpose:**

The purpose is explore an approach to acquire, analyze and report data concerning an organizational change initiative that combines knowledge generation and knowledge use, and contrast that with a method where knowledge generation and use is separated. More specifically, the authors contrast a participatory group workshop with individual interviews analyzed with thematic analysis, focusing on information about the change process and its perceived practical relevance and usefulness.

**Design/methodology/approach:**

Participants were managers responsible for implementing a broad organizational change aiming to improve service quality (e.g. access and equity) and reduce costs in a mental health service organization in Sweden. Individual interviews were conducted at two points, six months apart (i1: *n* = 15; i2: *n* = 18). Between the interviews, a 3.5-h participatory group workshop was conducted, during which participants ( *n* = 15) both generated and analyzed data through a structured process that mixed individual-, small- and whole-group activities.

**Findings:**

Both approaches elicited substantive information about the content, purpose and process of change. While the content and purpose findings were similar across the two data sources, the interviews described how to lead a change process, whereas the workshop yielded concrete information about what to do. Benefits of interviews included personal insights about leading change while the workshop provided an opportunity for collective sense-making.

**Originality/value:**

When organizational stakeholders work through the change process through a participatory workshop, they may get on the same page, but require additional support to take action.

## Background

The gap between what is known from research and what is done in practice is one of the most persistent challenges to improving quality of care, pointing toward translational gaps along the research-to-practice pathway ( Cooksey, 2006 ). Implementation science, defined as the scientific study of methods to promote the uptake of research findings into routine health care practice or policy ( Eccles and Mittman, 2006 ), is one response to this challenge ( Bauer *et al.* , 2015 ).

However, the time required for research to be implemented in practice is still too long. This has led to calls for more rapid approaches to health service and implementation research ( Riley *et al.* , 2013 ). As indicated in a systematic, integrative review of the concept of rapid implementation, there has been a steady increase in approaches aiming to speed up the research-to-practice transition by removing obstacles to the translation process in the last decade, with 20 of 24 articles being published after 2010, and ten after 2015 ( Smith *et al.* , 2020 ). Approaches range from pragmatic designs (e.g. pragmatic trials Riley *et al.* , 2013 ), and rapid data collection and analysis (e.g. Gale *et al.* , 2019 ), and use of pragmatic assessment strategies that include brief and actionable measures that produce feedback rapidly ( Battaglia and Glasgow, 2018 ), to rapid review methods ( Polisena *et al.* , 2015 ). Thus, these calls for rapid research ( Peek *et al.* , 2014 ) and rapid implementation ( Smith *et al.* , 2020 ) cover the entire spectrum, from a study's conceptualization to the reporting of findings. Reasons for using rapid approaches include reducing time and costs as well as increasing the amount of collected data by reducing time and resources spent on collecting it, improving efficiency and accuracy and reducing biases ( Vindrola-Padros and Johnson, 2020 ).

Nevertheless, these initiatives still adhere to a translational, pipeline approach to how research is turned into population impact ( Braithwaite *et al.* , 2018 ). The pipeline approach entails first conducting the research and then promoting its use in practice; for example, first ensuring that an intervention is efficacious and then disseminating and spreading it. The approach is motivated by the “rules of rigor” set up to ensure high internal validity and avoid erroneousness ( Geng *et al.* , 2017 ). Key characteristics of this approach are control, objectivity, rationality and predictability ( Wells and Mclean, 2013 ). Studied initiatives (e.g. interventions or organizational changes) are approached as episodic changes with initiatives that are predefined and static, with a clear beginning and end, and are independent of the context ( Weick and Quinn, 1999 ; von Thiele Schwarz *et al.* , 2016 ).

With the pipeline approach, knowledge is assumed to be developed in academia, by researchers who are viewed as the generators of knowledge, and then used elsewhere. Such approach has been criticized for assuming that knowledge generation and knowledge use, and knowing and doing, can be separated, and that knowledge is objective and impersonal ( Greenhalgh and Wieringa, 2011 ). The assumption of this so-called Mode-1 knowledge production ( Gibbons *et al.* , 1994 ) is reflected in terms such as *knowledge transfer* , *translation* and *implementation* and aligns well with summative approaches to evaluation, where the aspiration is to retain the intervention as it was developed to enable conclusions about its effectiveness ( Chambers *et al.* , 2013 ). Despite its advantages from a trustworthiness and internal validity perspective, approaching knowledge development in this way has also been criticized for contributing to the persistent gap between research and practice ( Greenhalgh and Wieringa, 2011 ; Harvey, 2013 ; Braithwaite *et al.* , 2018 ).

There are at least three reasons this knowledge development process may contribute to the research-practice gap. First, attempting to capture and separate the influence of the multitude of factors that affect the implementation process through control is, if even possible, cumbersome and time-consuming, given the complexity of the systems ( Braithwaite *et al.* , 2018 ). This may demand more resources than organizations are willing and able to use and may be misaligned with organizational demands for timely and actionable findings, limiting organizational stakeholders' willingness and ability to participate in research.

Second, the separation of research and practice communities may cause knowledge development to be driven by researchers and their interests rather than by the interests of the communities of users, such as health care professionals and health services ( Riley *et al.* , 2013 ). Such curiosity-driven research has been advocated to promote scientific discoveries that are applicable and available to the broader public rather than as a more limited benefit for a “special interest group,” such as a specific organization ( Estabrooks *et al.* , 2008 ). However, researcher-driven research may also entail researchers promoting their preferred approach regardless of its fit with organizational problems ( Wensing and Grol, 2019 ). Implementation research, which is explicitly concerned with getting a known solution into practice, may be particularly vulnerable to this risk if sufficient attention is not paid to how the solution fits an organization ( Reed *et al.* , 2019 ).

Third, the emphasis on objectivity, control and rigor may lead to a preference for methods that separate the variables of interest from the context of application. This can be viewed as an attempt to separate the research process from the change process. However, organizations are not passive recipients of research initiatives ( Nielsen, 2013 ) simply acting as an arena for academic endeavors ( Kristensen, 2005 ). In fact, they are more likely to be involved if they anticipate that they will benefit from the collaboration by, for example, improving organizational outcomes or managing change more efficiently ( von Thiele Schwarz *et al.* , 2020 ).

The attempt to separate the research process from the change process may be particularly futile for complex interventions consisting of multiple components that are not easily standardized, are carried out by multiple actors across units, organizations or systems, and change dynamically ( Hawe, 2015 ). In such changes, implementation becomes inseparable from organizational change, that is, “an initiative; which requires change to critical organizational processes that, in turn; influence individual behaviours; which, in turn, impact on organizational outcomes” ( Grieves, 2000 , p. 400).

What may be an implementation project for a researcher, thus, is likely to be an organizational change or a development project for the organization. With this, attempts to separate knowledge development from knowledge use may become futile, or at least impede the organization's ability to reap the benefits of collaboration by learning about, and improving, their change process as it unfolds ( von Thiele Schwarz *et al.* , 2020 ). Therefore, the development of rapid approaches needs to be complemented by approaches that facilitate *both* organizational change *and* are useful from a research perspective (e.g. contribute with data of sufficient quality for formal studies) ( Patton, 2006 ).

Approaches that address the objectives of both research and practice imply that instead of addressing knowledge generation and knowledge use sequentially, they are addressed in parallel. Such an approach to knowledge development has been labeled Mode-2 knowledge production ( Gibbons *et al.* , 1994 ). With Mode-2 knowledge production, the aspiration is to form non-hierarchical relationships between researchers, policymakers and practice to address a research problem in a specific context and to achieve knowledge that is both socially and academically robust ( Estabrooks *et al.* , 2008 ).

The recently promoted fourth research paradigm ( Rapport and Braithwaite, 2018 ) is built on similar propositions to move from siloed, a-synchronized approaches to those that are characterized by co-design and co-creation, shared intentions, real-time acquisition, analysis and data sharing. Practically, this may entail integrating data collection and data analysis for implementation research into organizational development, making the research activities part of organizational activities to promote change.

Yet, there is a shortage of practical methodological approaches that can meet the dual needs of being useful for research *and* practice. This includes data acquisition that is rapid but yields trustworthy *and* actionable data that stimulate reflection and learning among participants.

Co-created program logic (COP) is a reinvention of a participatory workshop methodology originally designed for curriculum development ( Savage, 2011 ). COP's purpose is to facilitate a shared understanding of the change purpose, content and process among organizational stakeholders while simultaneously informing implementation, evaluation and research, thereby being mutually beneficial for organizational change *and* research purposes ( von Thiele Schwarz *et al.* , 2018 ). More specifically, COP aims to (1) provide designers and evaluators (researchers) with information to inform design as well as its implementation and evaluation; (2) provide organizational stakeholders with actionable information about the purpose, content and process of change while promoting a shared understanding of change and sense-making ( Weick, 1995 ); and (3) lay the groundwork for an evaluability assessment by creating consensus between researchers (evaluators) and organizational stakeholders about the change, thereby addressing potential obstacles to acceptance of evaluation results up-stream ( Leviton *et al.* , 2010 ).

In contrast to traditional methods, where the researcher collects data, conducts analysis and reports the results, COP participants are actively involved both in gathering and analyzing information. Active participation is a central tenant of COP. Participation has been described as the holy grail of improvement work ( Wensing and Grol, 2019 ); yet, it can mean many things ( Abildgaard *et al.* , 2020 ; von Thiele Schwarz *et al.* , 2020 ; Reed *et al.* , 2019 ). The COP approach to participation is inspired by “the wisdom of the crowd,” which involves the assumption that a group may collectively understand a phenomenon, such as an organizational change, although it is only known in parts by the individuals. Thus, the workshop is designed to promote sharing and interaction between participants to bring together the pieces of the organizational change puzzle spread out between the individuals.

During the workshop, the group shifts between working on individual tasks, in sub-groups with different parts of the problem, and the whole group, guided by four principles: (1) minimize the suppression of individual perspectives (i.e. support *independence* ) by allowing everyone to make their perspectives known; (2) encourage *diversity* by inviting people who are both close and more distant to the change to participate; (3) *decentralize* by enabling participants to work on different tasks or aspects of a problem; and (4) *aggregate* by bringing the distributed efforts together to form a collective understanding ( Hosseini *et al.* , 2015 ). Specifically, individuals bring their understanding of the organizational change for the collective to put together in a program logic, explicating implementation and end-outcomes from the descriptions of the core components, the purpose of the change and the actions needed to bring about the change, such as implementation activities ( Olsen *et al.* , 2012 ; Rogers, 2008 ).

Also, the workshop is designed to stimulate reflection-in-action, that is, not only to generate data but also to notice the process and reflect on the data, including the variation between individual and what that may represent ( Schön, 1991 ). This acknowledges that to drive change in organizations, a rational “strategic grand narrative” of the planned change may not be sufficient ( Grieves, 2000 ). It needs to be complemented by reflective awareness among participants. In all, COP breaks with the roles and division of work that often characterize the interaction between research and practice and promotes reflection and action *by and with* , not *on* , practice ( Cornwall and Jewkes, 1995 ).

## Aim

The aim of this study is to explore an approach to acquire, analyze and report data concerning an organizational change initiative that combines knowledge generation and knowledge use (a participatory group workshop), and then contrast that with a method where knowledge generation and use is separated (interviews). In this, we aim to address recent calls for ways for implementation research to be more useful for participating organizations. More specifically, we contrast a participatory group workshop with individual interviews analyzed with thematic analysis, focusing on information about the change process and its perceived practical relevance and usefulness.

## Method

### Study design

This is a mixed-method study using a comparative exploratory approach with interviews conducted in May–June 2019 (i1), a workshop in August 2019 (w1), and a second round of interviews conducted in September–October 2019 (i2).

#### Setting and organizational change context

The study took place at the Division of Psychiatry and Disability in Region Sörmland, Sweden, which provides services related to mental health, neurodevelopmental disorders and developmental disabilities to a population of about 300,000, both children and adults. The division employs about 900 people, including psychologists, physicians, and nurses, at ten geographically dispersed units.

In 2018, the division faced three main challenges related to neuropsychiatric assessments which acted as drivers of change. First, there was no standard protocol for the assessments, leading to unwanted variations in services and quality and inequalities in service provision. Second, there was a shortage of skilled staff across the organization, and third, there had been a rapid increase in individuals seeking neuropsychiatric assessment. This situation had led to long waiting times for patients and a 600% increase in costs from 2016 to 2018, due to external costs that resulted from the region being obligated to procure services from external providers if patient waiting times exceeded 90 days. A formal decision regarding the purpose and core components of the change was stated in organizational documents (see Table 1 ).

A research-practice collaboration was established in spring 2019 and founded on an existing collaborative agreement between Mälardalen University and the surrounding local communities. The goal of that collaboration was both to support the organization in its change efforts and to investigate the scientific and practical value of the methods used to study change.

### Participants and recruitment procedures

The entire management team responsible for leading the change was invited to participate. This included unit and operation managers and the head of the division (14 at i1 and 16 at w1 and i2) and administrative support, i.e. a HR specialist, controller, business developer and project leader (three at i1 and four at w1 and i2). The participants were informed about the project by the researchers through a video recording and a coordinator in the region scheduled both rounds of interviews for those wishing to participate. The interviews were conducted by one of two interviewers and took place face-to-face at a place convenient for the participant. Informed consent was obtained before the first data collection. Participants were invited to the workshop through a letter from the researchers attached to an email from the head of the division. The workshop was described as an active afternoon, with change management/improvement work as a theme in which the group and researchers would work together to create a common understanding of change (i.e. purpose, content, and process). All but one invited participated in i1 ( *n* = 15), 15 of 20 in w1 (12 of whom had participated in i1, three that had joined the organization between i1 and w1), and 18 of 20 in i2. The project was reviewed by the Swedish Ethical Review Authority and found not to need ethical approval (DNR 2019-03942).

### COP – participatory method for data collection and analysis

A participatory COP workshop was conducted in a single 3.5-h session. The process followed five steps ( von Thiele Schwarz *et al.* , 2018 ) (see Table 2 ). The workshop included a mixture of individual and collaborative work, supporting wisdom-of-the-crowd principles of independence (i.e. individual work), decentralization (by dividing the group into sub-groups and pairs; step 3a and 4b) and then aggregation (i.e. entire group tasks).

Reflection-in-action was promoted by asking participants reflective questions, such as about various ways of understanding the change. The material produced during the workshops – unprocessed Post-its (steps 1b and step 5) as well as Post-its that were sorted (step 3a) and matched (step 4b) – was compiled and transferred to an Excel file. The analysis from steps 3a and 4b is reported in the Results section. The Excel file was also shared with the organization the day after the workshop as a basis for their continuous improvement efforts.

### Evaluation questionnaire and analysis

The workshop's relevance and usefulness were evaluated through a short questionnaire handed out at the end of the workshop. The workshop's overall appraisal was assessed using the Generic Workshop Appraisal Scale (WASC) ( Fridrich *et al.* , 2020 ). Participants were asked to rate how they perceived the workshop on a 10-item scale with pairs of adjectives covering five facets: comprehensibility, relevance, novelty, activation and valence (e.g. unimportant – important; well-known – novel). The items were rated from 1 to 10, with the negative adjective as 1 and the positive as 10.

Appropriateness, the perceived fit, relevance or compatibility of an intervention, including how much it follows the provider's skill set, role or job expectations ( Proctor *et al.* , 2011 ), was assessed by two items for the degree to which participation in the workshop was an efficient use of the participants' time and competence, from “not at all” (1) to “extremely” (10). Using the same response format, participants indicated the degree to which the workshops had increased their shared understanding of the change, their own understanding of the change and their understanding of own role. Data were analyzed with descriptive statistics.

The questionnaire ended with three open-ended questions that asked what from the workshop worked well, what could be done differently and what the participants personally took with them from the workshop.

### Interview description and analysis

The interviews used an exploratory approach with open-ended questions developed with the head of the division and the business developer to ensure that the findings were useful for the organization and research. The questions aimed to cover the current organizational change, including the purpose, content, and process of change, focusing on respondents' experiences and reflections. In contrast to the workshop, the intention was to provide a free space to talk about the change, learnings, and one's change leadership. Lastly, the experience of participating in the interview was explored. The second interview followed up on questions from the first interview and discussed the experience of participating in the workshop and interviews. The interviews lasted 44–68 min, and all were audiotaped.

The data analysis was guided by Braun and Clarke's six phases of thematic analysis ( Braun and Clarke, 2006 , 2020 ). TA was chosen because the aim of the study called for a flexible method that, on the one hand, would allow findings from the interviews to be compared to findings from the workshop and, on the other hand, would ensure that the analyses reflect the richness of the interview data.

Specifically, we used a combination of codebook TA and reflexive TA to enable topic summaries of what the three aspects of change entailed according to interviewees, making them comparable to the workshop's outcomes (i.e. codebook TA) while capturing “the patterns of shared meaning” (i.e. reflexive TA) ( Braun and Clarke, 2020 ). In the Results section, the findings from the codebook TA are reported as topics, and the findings from the reflexive TA are reported as themes.

During the first step, the data familiarization and interview transcripts were read and organized according to the three aspects of change (i.e. purpose, content and process of change) by color-coding relevant sections. This was done by each interviewer. The subsequent steps were taken by one of the interviewers, who was not involved in the COP workshop. The interview transcripts were coded inductively without trying to fit them into any pre-existing coding frame. Next, the codes were combined in two ways: by summarizing them in topics corresponding to the purpose, content and process of change, respectively (i.e. codebook TA), and by combining codes that reflected shared patterns within each change aspect, thereby generating initial themes through a reflexive TA.

The significance of a theme was determined by how well it captured something beyond the topic summary, allowing common themes across respondents to be collated and described in a meaningful way. Thus, the themes were mainly identified at a latent or interpretative level, going beyond the data's semantic content. The initial themes were then further developed by rereading the transcripts and initial codes and refining the themes so that they conveyed the pattern in the data in a meaningful way. Finally, the topics and themes were refined, defined and named through discussions between all authors, including rereading the interview transcripts and codes.

## Results

The following section first present the findings concerning what information about the change process that the two methods conveyed about the purpose, content and process of change. The findings from the workshop are based on analyses made by the participants as part of the workshop, and the interview findings are based on the TA (i.e. codebook and reflexive) done by researchers. The final section presents the interviews' and workshop's perceived relevance and usefulness.

### Purpose: what are the aspired results of the change process?

Table 3 presents the purpose and content of the change from the COP workshop and the corresponding topics from the codebook TA analysis of the interviews. A comparison of the findings from the workshop and the interviews indicated that the three main topics found in the interviews (i.e. *better finances, improved patient flow* and *improved patient support* ) were also reflected in the findings from the workshop.

For example:

To remove obstacles – make it flow. (COP – Smooth Patient Pathways)

That we achieve a better structure, that we use the resources we have in a way that leads to … more and better care, more effective care. That we get the accessibility in order, we have to be better at taking care of those that need us. (interviews – improved patient flow)

The workshop included four additional topics. Of these, *a more cohesive division* was addressed most frequently in the interviews, albeit not to the degree that it was perceived as a major topic. Thus, the workshop analysis resulted in more topics than interviews. Interview analysis (i.e. reflexive TA), on the other hand, generated two themes that provided an additional perspective on how the purpose of the change was understood.

The first theme was “ *purpose depending on position* ” and reflected that there was great variation between participants depending on, for example, their role in the organization. The second theme was *“purposes differing in importance* ,” something that was not immediately apparent from the workshop analysis. The high cost of out-of-county assessment was described as triggering the change, making better finances appear as the most important objective. However, this was also because achieving it was viewed as critical for realizing the other purposes.

It is partly a matter of reducing the number of patients opting for assessments out-of-county because it is a huge cost. So, overall, it's about that. (Interview – Better finances)

### Content: what are the core components of the change process?

Overall, the descriptions of core components from the workshop and interviews were similar, focusing on the establishment of a new unit that specializes in assessments that the change would include addressing who does what, while using briefer, more time-efficient assessment methods and new patient pathways. For example, the workshop theme “ *new ways of working* ” included notes about the need to reduce variations on how assessments are done and work more effectively.

The focus for employees needs to shift from we are ‘used to working like this’ to ‘what is best for the patient.’ e.g. a faster path to diagnosis and treatment. (COP – new ways of working)

Topics also brought up in the workshop included *recruitment* , new routines for *waiting lists,* and a need to shift focus to *activities after assessment* . In addition, the participants also brought up components relating to the change process, such as *participation* and *communication* , as core elements of change.

The reflexive TA of the interviews indicated two central themes that summarized the way participants understood the core components of change: *Agility – letting the goal direct actions* and *allowing variation* . Agility reflects an aspiration to focus on the goal rather than on the activities, letting the content of the change evolve. Allowing variation reflects room for variation in the process and content, depending on the type of operation and position that is based on local needs.

In line with this, the interviews again indicated that managers' views on what the change entailed varied, between each other and over time. Whereas it was common to describe the change as multifaceted and including a variety of activities varying over time and units, there were also descriptions characterizing the change as simply being about establishing a dedicated unit for neuropsychiatric assessments and with little relevance for their own unit.

I think there are many different parts. But for me, the most important thing will be how our unit can improve processes and flows. That will be most important for me. Then I'm positive about the other parts, but they may not be the ones I need to do so much about. (interview)

### Process of change – workshop

The COP workshop's participants produced 71 notes in response to the question, “What needs to be done to make the change happen?” and assigned them to the most appropriate of the eight roles/functions they had identified. The number of tasks per role varied from two (operations managers and head of the division, respectively) to 13 (new unit). Some tasks were assigned to more than one role. For example, all managers were expected to monitor and provide feedback, be present, and make sense of the change. Unit managers working closest to staff should also motivate staff to engage in the change process and retain and recruit staff. Higher management's tasks included providing clear direction, creating cohesion and coordinating activities. Employees were expected to develop and implement new methods for neuropsychiatric assessments. The new unit's comprehensive list of activities included advertising the new unit, matching competence to tasks, and trying new technical solutions. The supporting roles, such as the business developer, were assigned tasks that focused on analysis and planning.

Examples of Post-it notes are as follows:

Get employees involved in the change process. (COP, first-line manager)

Clear overall action plan (COP, division management team)

Look at ‘work shifting,’ who does what in the assessment process. The right competence in the right place. (COP, new unit)

Develop templates for assessment reports so not everyone has to develop their own.’ (COP, employees)

### Process of change: interviews

**Figure 1:**
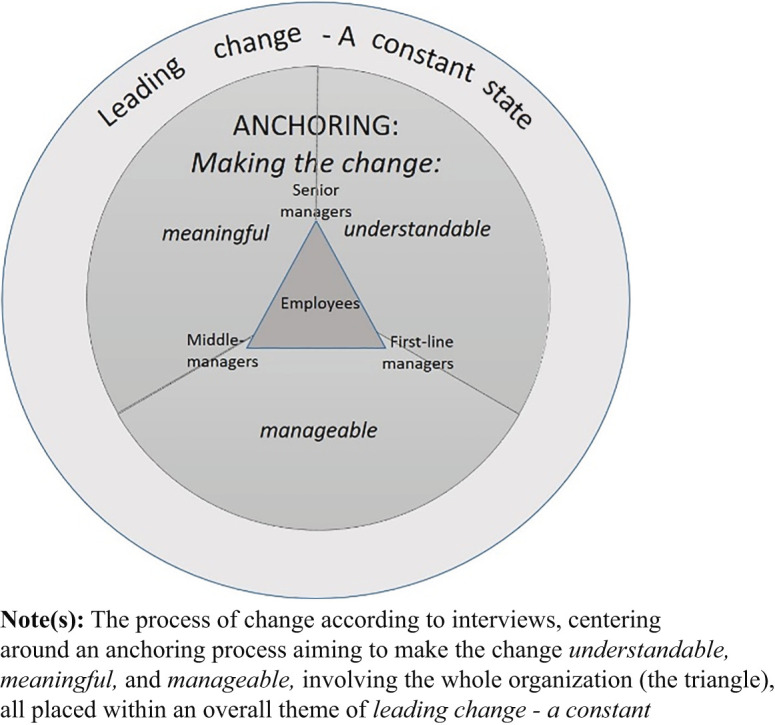
Themes from interviews describing the change process

In contrast to the workshop's focus on activities and roles, the interviews painted a broader picture of the process of change, conveying the participants' personal reflections on leading change. Thus, it provided a perspective on how participants reasoned about *how* the change process was approached. The themes summarizing this, and how they are interrelated, are presented in Figure 1 .

The core of the change process was *anchoring* , a practice focusing on making the change *understandable* , *meaningful* and *manageable* . *Anchoring* involves an ongoing process of sense-giving to get everyone on board and moving in the same direction. Anchoring is more than conveying information:

… how valuable it is, to gather ideas. My first thought was to get them to go along with my idea, but then it got much better, because they came up with additional ideas. That was good.

Yet, sense-giving requires sense-making: managers need to understand the why and what themselves in order to ensure that others understand the change and find it meaningful and manageable.

That is probably what happens: If I don’t have full overview and am supposed to pass on information, and get questions, then it becomes fuzzy … or I could immediately say that I don’t know. I can certainly do that, but if we do that all the time, it will not be so good or inspire much confidence. So, it is crucial to be informed yourself and understand what we do and why, and the different steps.

Figure 1 also shows a theme surrounding the anchoring process: *leading change – a constant state* . This particular change was one of many and its importance varied according to different managers depending on how their unit was affected, what else was going on, and how this change compared to other changes they had been involved in.

But then I think there have been changes all the time. But it all happens … it is so rare to say “now this is change work”. But of course it is. You adjust a little here and sometimes you change a lot and make substantial changes, so it has been ongoing … But I think there have been situations when I have not realized that we are actually making a rather big change, until maybe after a while …

### Perceived relevance and usefulness of the workshop and interviews

The perceived relevance and usefulness of the two approaches were evaluated using questions at the end of the interviews and in writing at the end of the workshop.

#### The workshop

**Figure 2:**
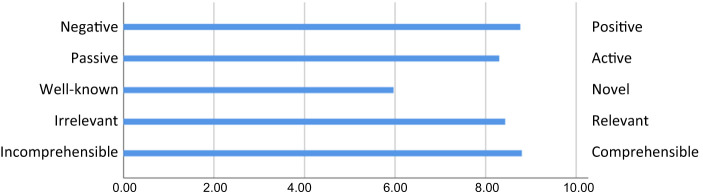
The COP workshop’s evaluation ratings

Figure 2 illustrates how the workshop was appraised and indicates that the workshop was overall seen to be positive, comprehensible, active, and relevant, but not particularly novel. In terms of the appropriateness of the workshop, participants judged it to be an efficient use of time ( *M* = 7.0, *SD* = 1.6) and competence ( *M* = 6.6, *SD* = 2.3) overall but there was great variation between participants (3–9 and 1–9, respectively). Similarly, the workshop was judged as having increased shared understanding overall ( *M* = 6.9, *SD* = 1.7, min-max 4–10) with slightly lower ratings and greater variations concerning the degree to which the workshops had increased a participant's own understanding of the change ( *M* = 5.7, *SD* = 2.7, min-max 1–9) and understanding of their own role ( *M* = 5.7, *SD* = 2.8, min-max 1–10).

The written evaluation immediately after the workshop and the interviews that took place two to three months later both conveyed that participants overall liked the structured, participatory workshop approach that combined individual reflections, rapid documentation on post-it note, and joint analysis.

I think it's great that people sit and reflect on their own first and that there is some sort of common reflection afterwards. And categorizing makes people think “But what did I really mean?” or “what do I think this person means by this” and “You could think like that too.” (interview)

However, not all participants favored the participatory, active approach. Some also expressed that they would (also or instead) have liked the researchers to share their knowledge of change process.

My expectations were high, and I thought we would hear a little more about ongoing research or experiences worth considering. (interview)

The managers' involvement in the change differed, influencing their perceptions of the relevance and usefulness of the workshop. Their evaluations were also influenced by whether they viewed the workshop from an individual or collective perspective. Some new managers felt that there was a lot to take in and their comfort with being novices varied; overall, they felt that they learned a lot. Some that were deeply involved in the change felt that the workshop offered little new information from an individual perspective and suggested that it would have been more useful earlier in the process. Conversely, both managers heavily involved in the change and those in more peripheral positions saw the value in it from a collective perspective.

I think we came quite far considering … it's so interesting because we are still from different units. There was a fairly clear consensus concerning the (post-it)-notes sure, different formulations but it is the same core all the time. It was interesting to see. No matter what your conclusion is, you have, I think, created a greater affinity among us. (interview)

The benefits lay in bringing together the experienced and less experienced and those more central and less central to the change. The participants indicated that the workshop brought different ways of understanding the change forward, helping them to identify what they had a shared understanding of, and where there were views that did not converge.

It was interesting to hear the persons describing the change during the workshop, describing it as only being about the new unit. And sure, if it is realized – excellent! But that really is not all. … There are other parts too, that has nothing to do with the new unit. (interview)

The evaluation of the COP workshop was overall more positive judging from the data immediately after the workshop than from the interviews held two to three months later. Some participants expressed at that time that they lacked feedback from the workshops, indicating that nothing had happened since, making the workshop appear to be an isolated event.

*Interviews.* The evaluation of participation in interviews revealed that participants had lower prior expectations on participating in interviews than they had on the workshop. While a few participants primarily thought of their participation as a contribution to research, most were positively surprised that participating in interviews gave them an *opportunity to reflect* on their own leadership and change processes in general and the current change process in particular.

Sometimes it's good to just talk about changes; were you are and what you have done so far, what is successful and what the challenges are. (interviews)

## Discussion

Inspired by the movement toward rapid methods in implementation research and Mode-2 knowledge production, this study aimed to describe and contrast the information gained from two approaches to data acquisition, analysis, and reporting. The findings show that, overall, a participatory workshop where the participants generated and analyzed data and a TA of individual interviews conveyed similar information about the content and purpose of the change. However, more concrete information about who should do what was gleaned from the workshops and more reflective information about how to manage change from the interviews. In addition, workshops stimulated collective sense-making while interviews stimulated reflection on one's own role and journey; the value and relevance of the methods were affected by individuals preference for and preparedness to be part of a participatory knowledge generating process.

The workshop had dual aims of contributing information of sufficient quality to be used in research while also serving a purpose for the participating organization. The findings showed that the rapid analysis made by participants concerning the purpose and content of the change generally matched the results from the TA as well as the organization's own documentation. Although this does not automatically imply high quality, it offers some reassurance that methodological approaches that put relevance and change in practice in the forefront does not need to be inferior to other methods ( Wensing and Grol, 2019 ). Thus, rapid analysis done by participants rather than experts does not automatically reduce quality, an issue that has previously been raised due to concerns regarding limited capacity (time and money) and/or competence to conduct qualitative analysis ( Burgess-Allen and Owen-Smith, 2010 ). The findings support previous indications that, from a research perspective, even a very brief workshop session can yield data that can guide evaluation and offer insights to improve implementation by, for example, realizing what processes are perceived as most important for successfully managing change ( Hasson *et al.* , 2018 ; Augustsson *et al.* , 2017 ; von Thiele Schwarz *et al.* , 2018 ).

The goal of the workshop was to provide the organization with actionable information while also promoting a shared understanding of the change. However, little overt action related to the workshop took place in the two months following the workshop. Therefore, the overall positive view of the workshop shifted somewhat between the end of the workshop and the interviews two months later. The perceived lack of action may be due to the timing being off, illuminating the challenge of timing an initiative like this to a change process that is dynamic and change over time. That the COP approach did not include guidance for the organization on how to proceed, which may also have impeded actionability. It is also possible that there *had* been activities, but that this had not yet been noticed in the organization, or not connected to the workshop. Thus, the perceived lack of action taken may represent an implementation problem. Further research is needed to understand how findings from these kinds of workshops can be implemented in practice.

The findings show that the workshop indeed supported shared sense-making. Interviews, in contrast, prompted more personal reflections and individual sense-making. Although the workshop was designed to stimulate personal reflections and encourage “reflection-in-action” as well as collective understanding, the latter seemed more successful than the former, and participants' individual reflections were not prompted in the same way as in the interviews. This implies that some will “give more than they get” from the exercise and tolerance for that may differ. Even though group-based activities cannot be adapted to individual needs as much as interviews can, acknowledgment of the variation within the group and justification for the process may increase the acceptance among those individuals that give more than they get. Otherwise, the workshop may come at the cost of over-emphasizing collectivization and pushing participants to consider communalities at the expense of their individual perspective while interviews may over-emphasize individuality and push participants to concentrate on their own journey rather the joint one. In this, the workshop may contribute to strengthening the collective and the interviews the individual.

One aspect that may require further attention to optimize the utility of the workshop is the roles of researchers and practitioners. Previous research emphasizes the resistance to change in the research community ( Kristensen, 2005 ; von Thiele Schwarz *et al.* , 2020 ), and the challenges that researcher may face when they engage in Mode-2 knowledge production in an academic context that rewards Mode-1 research ( Estabrooks *et al.* , 2008 ). Our findings show that views, preferences and expectations of practice partners, too, need to be considered for a shift from Mode-1 to Mode-2 knowledge production to occur. Data from both the workshop and interviews showed that some participants explicitly requested a more traditional approach where practitioners are recipients of knowledge and researchers act as experts, do the analysis, and report back as opposed to practitioners being agents in their own change and co-creating knowledge, as stipulated by a Mode-1 knowledge production process. For example, even though participants had done the analysis themselves as part of the workshop, and had all the documentation, some would have liked the researchers to be more active, providing feedback etc. A hybrid approach wherein a researcher takes a more active role, for example by sharing their own reflections on the process more, may be one way forward.

### Methodological consideration

The COP workshop was run by the researchers, one of whom was part of developing the workshop. In psychotherapy, having a developer involved has been shown to inflate the effectiveness of interventions (an experimenter allegiance effect) possibly due to their familiarity with the method and their passion for it ( Dragioti *et al.* , 2015 ). However, the workshop was highly structured, leaving less room for workshop leaders to influence the process beyond the skills required to engage people in conversations and prompt reflections on observations and learnings.

In terms of the analysis of workshop data, the time participants spent on analysis during the workshop was restricted, with the goal of arriving at a representation of the collective understanding of the change process rather than exhausting all iterations and arriving at a fully saturated solution. This represents a trade-off between specificity and efficient use of participants' time. The workshop analysis, therefore, should be considered a first iteration that could be further refined in subsequent sessions. Concurrently, the analytic approach, whereby several individuals are involved and participants initially work together in silence, also has advantages such as reducing some of the potential biases in qualitative research, including that individuals' preconceptions can influence the results. An advantage from a research perspective is that the researcher receives an analysis that is independent of them. Thus, the COP workshop may be used to validate findings from a traditional research approach.

Most participants took part in both interviews and the workshop. Even though participants were asked to reflect on each approach separately, it is probable that participating in one influenced the experience of the other. For example, the first interview may have made participants more prepared for the workshop, or lessen the perceived need for it. Yet, having experienced both may also have accentuated the differences between the methods. Moreover, the findings should be interpreted in light of the participants being accustomed to using participatory approaches, reducing its novelty for better or worse. Overall, the appropriateness and usefulness of methods may be influenced by contextual factors such as the participants’ previous experience, expectations and concurrent activities.

To avoid contamination between the two approaches, the reporting of workshop findings and the analysis and reporting of the interview data were done independently and by different persons. However, for practical reasons, about half of the interviews was conducted by a researcher that was also present for but not leading the workshop. Although this may increase the risk of response bias by, for example, avoiding reporting negative attitudes of the workshop to the interviewer, there were no observed systematic differences depending on who conducted the interviews.

## Conclusions

While there has been an increase in number of methods aimed to speed up the implementation research process, most methods still primarily reflect a traditional take on the knowledge development process (first research, then practice) by separating the knowledge generation process from knowledge use. Concurrently, a scholarly discourse about alternatives where knowledge generation and use are intertwined is growing in the field of implementation science. In addition to the epistemological implications, a transformation of the knowledge development system also has very practical ones. This study explored a one-session 3.5-h workshop where practitioners elicit information about a change process and analyze it together. In addition to showing, through triangulation, that the workshop yields information that may be used for research purposes, this study also demonstrated that this format promotes collective sense-making for participants and provides them with concrete, *actionable* information. However, that does not mean that the obtained information is automatically *acted* upon. Researchers are not the only ones with assumptions regarding how research is done – practitioners have them too and change is needed across the whole research-to-practice pathway if true transformation is to occur.

## Figures and Tables

**Table 1:** Purpose and content of change as stated in organizational documents

Purpose of change	Content of change
Equitable care across the units High availability of services Good quality and respectful treatment of patients Efficient care adapted to patient needs Clear processes leading to more patient contacts Fewer patients choosing external providers	Create a new position, to guide patients to appropriate level and type of care Improve patient flows at all units; establish a dedicated neuropsychiatric assessment unit Offer extra shifts on evenings and/or weekends to work off queues Explore instituting referral requirements Create a communication plan (inwards and outwards) New procurement of external assessments

**Table 2:** The five steps in the COP-workshop

	Aim of step	Question	Individual activity	Group activity
Step 1a	Explicate the aspired results of the change process; the purpose	What will success look like?	Responses on post-it notes, one note per response	
Step 1b^a^	Explicate the unintended consequences of the change	What unintended consequences could the change lead to?	Responses on post-it notes, one note per response	Sort out notes with probable and/or relevant consequences
Step 2	Identify change components	What are the core components of the change process?	Responses on post-it notes, one note per response	
Step 3a	Create and name themes summarizing the purpose (step 1a, subgroup 1) and content (step 2, subgroup 2) in about 6–10 themes	How can the notes be grouped in a way that is meaningful to you? How does that converge with others' ways of understanding?	Reflect on individual understanding, move post-its in silence	Sort notes together in silence. Then discuss what characterizes each theme
Step 3b	Accumulate sub-group knowledge and individual reflections for the whole group	Were you alike? Where do views differ?		Reflect together on where there is agreement and where there is not as well as what you are learning
Step 4a	Identify what needs to be done (process of change)	What needs to be done to make the change happen?	Answers/reflections on post-it notes, one note per answer	
Step 4b	Assign what needs to be done to the relevant players in the organization	Who needs to do what for the change to be successful?	Work in pairs, decide central roles and assign each task to the relevant role	Reflect together on where there is agreement, where there is not, and what you are learning
Step 5^a^	Identify factors/elements that would enable the managers to succeed in their role	What do the managers need to succeed with their tasks/responsibilities?	Answers/reflections on post-it notes, one note per answer	

**Note(s):** ^a^The information was not further processed during the workshop. It intended to ensure reflection on unintended consequences and need for support; the group was handed back the documentation to utilize in their continued change efforts

**Table 3:** The change *purpose* and *content* according to workshops (as analyzed by participants) and interviews (thematic analysis by researcher)

	Data domains based on participants analysis during COP workshops	Data domains based on thematic analysis of interviews
Purpose: What are the aspired results of the change process?	- *(Better) finances* - *Smooth patient pathways* - *Satisfied patients* -A more cohesive division -Good work environment -New ways of working -Pride in our region's healthcare	- *Better finances* - *Improved patient flow* - *Improved patient support*
Content: What are the core components of the change process?	- *New unit* - *Resource allocation* - *New ways of working* - *Resource allocation/new ways of working* ^a^ -Participation -Communication -Framework for change -Willingness to change -Recruitment -Waiting lists -Activities after assessment	- *Establish a dedicated neuropsychiatric assessment unit* - *Look over work processes (what, how, when, by whom)* - *New assessment methods* - *New patient pathways* -Agility; letting the goal direct actions -Allowing variation in process and content depending on type of operation and position

**Note(s):** ^a^A single note (teamwork, new assessment methods, new personal categories) that was put in a single category
